# A Rare Case of Acute-Onset Spastic Quadriparesis Caused by a Chondroma of the Cervical Spine

**DOI:** 10.1155/2019/3131628

**Published:** 2019-05-23

**Authors:** Kaori Momota, Toshihiko Nishisho, Ryo Miyagi, Shunichi Toki, Kazuta Yamashita, Fumitake Tezuka, Yoichiro Takata, Toshinori Sakai, Akihiro Nagamachi, Toru Maeda, Koichi Sairyo

**Affiliations:** ^1^Department of Orthopedics, Institute of Biomedical Sciences, Tokushima University Graduate School, Tokushima, Japan; ^2^Department of Orthopedics, Takamatsu Municipal Hospital, Takamatsu, Japan

## Abstract

Chondromas are benign cartilaginous tumors that occur very rarely in the spine. Moreover, chondromas of the extraskeletal origin are also very rare. In this case report, we describe an extremely rare case of chondroma arising from the ligamentum flavum in the cervical spine. A 67-year-old man presented to our clinic with acute-onset spastic quadriparesis. We performed emergent magnetic resonance imaging and found an epidural mass in the right ligamentum flavum at C4-C5. The acute-onset presentation was suspicious for intraligamentous hematoma in the ligamentum flavum at this level. We performed emergency decompression surgery and en bloc removal of the epidural mass with the right C4 and C5 lamina. The lesion had the appearance of cartilaginous tissue rather than hematoma. Based on the histological investigation, the final diagnosis was intraligamentous chondroma of the cervical spine. The quadriparesis improved postoperatively, as did the results of manual muscle testing in the affected area, and he was able to resume walking independently with a cane. At the one-year follow-up, the manual muscle testing results were almost normal. Surgeons should keep in mind the possibility of benign tumors including chondroma of the cervical spine when a patient presents with acute-onset quadriparesis.

## 1. Introduction

Soft-tissue chondroma is a benign mesenchymal neoplasm that contains cells with a chondrocyte phenotype, secretes cartilage matrix, and arises in extraosseous and extrasynovial tissues [1]. Although these tumors can arise at any age, most patients are middle-aged (mean, 34.5 years) [2, 3]. Two-thirds of these tumors occur in the fingers [3–5], and the rest arise in the hands, followed by the toes and feet. Chondromas that originate in the trunk or the head and neck region [6] are uncommon, and those that have their origin in the spine are very rare. In 2015, Byun et al. reported a case of cervical chondroma and reviewed the literature [7], which at that time contained only 10 cases. More recently, in 2018, Inoue et al. reviewed chondroma in the cervical region and identified 16 cases [8]. In most cases, the lesion was found because of paresis.

Most chondromas are solitary and present as a painless mass in the soft tissue around tendons and joints. Chondromas arising from ligamentous tissue are rare, with only one case report involving the knee reported in the literature [9]. In this report, we describe a patient who presented with acute-onset spastic quadriparesis and was found to have a chondroma in the cervical region that was compressing the spinal cord. The tumor was located in the ligamentum flavum in the cervical spine, which is apparently quite rare. This study was approved by the Ethics Committee of Tokushima University.

## 2. Case Report

A 67-year-old man presented to our clinic with acute-onset spastic quadriparesis. During the previous 2 years, he had been under follow-up at 3-month intervals in our clinic following decompressive laminectomy for lumbar spinal canal stenosis and fusion surgery for ossification of the ligamentum flavum of the thoracic spine. In April 2016, he suddenly noticed quadriparesis. He could not stand or walk, but did not present to our clinic until his scheduled follow-up visit 2 weeks later. Manual muscle testing (MMT) confirmed right-dominant quadriparesis (Table 1). Patellar and Achilles tendon reflexes were hypoactive, suggesting likely spinal canal stenosis and ossification of the ligamentum flavum. He had a spastic, broad-based steppage gait and needed a walker for ambulation.

**Figure 1:**
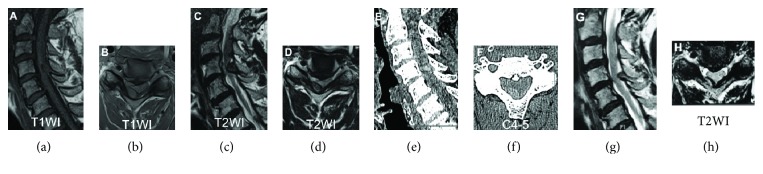
Magnetic resonance images (MRIs) and computed tomography scans (CTs) acquired at the time of acute onset of quadriparesis (a-f) and MRIs acquired 2 years earlier the quadriparesis (g, h): (a) sagittal T1WI, (b) axial T1WI, (c) sagittal T2WI, (d) axial T2WI, (e) sagittal CT, (f) axial CT, (g) sagittal T2WI, and (h) axial T2WI. Images at the onset shows a mass in the spinal canal at C4-5 on the right that is compressing the spinal cord. CT indicates that the mass did not contain osseous tissue. On the other hand, MRIs acquired 2 years earlier to investigate neck pain show that the tumor was present at that time but was not large or compressing the spinal cord.

**Figure 2:**
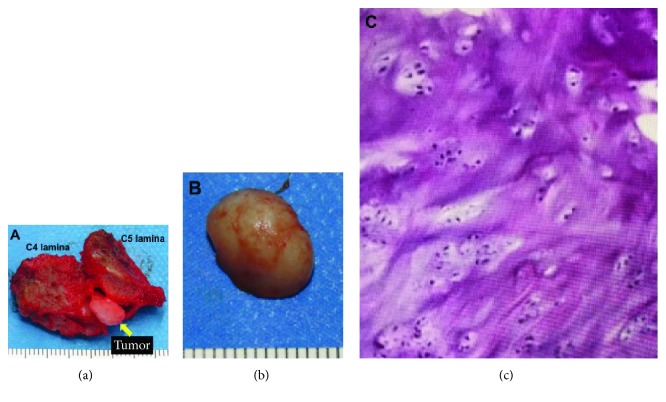
(a, b) Macroscopic features of the tumor specimen with the cervical lamina (the smallest division of the scale is 1 mm). (c) Histological findings from the resected tumor revealed cartilaginous tissue with no atypical cells (hematoxylin-eosin staining).

**Figure 3:**
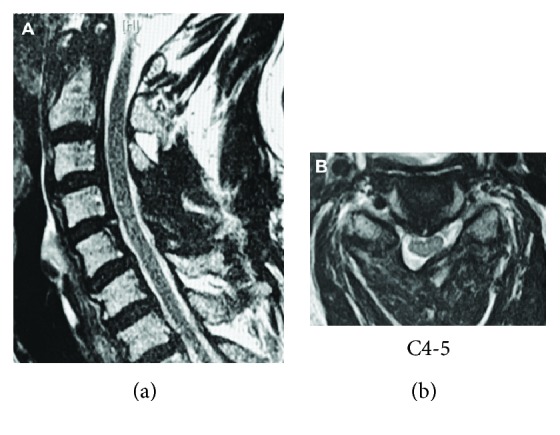
Postoperative MRIs confirm decompression of the cervical spinal cord. (a) Sagittal T2WI. (b) Axial T2WI.

Emergent magnetic resonance imaging (MRI) revealed an epidural mass in the right ligamentum flavum at the C4-C5 level (Figures 1(a)–1(d)). The mass was isointense in T1WI and low to isointensity in T2WI. Computed tomography (CT) scanning confirmed that there was no ossification in the mass (Figures 1(e) and 1(f)). Given the acute and rapidly deteriorating clinical presentation, we thought this case was intraligamentous hematoma in the ligamentum flavum in the cervical spine. However, cervical MRI scan that had been acquired 2 years earlier to investigate neck pain (Figures 1(g) and 1(h)) revealed that the tumor was present at that time but was smaller and not compressing the spinal cord. Therefore, the differential diagnosis was soft tissue tumor as well as hematoma. We performed emergency decompression surgery with removal of the epidural lesion. We removed the mass en bloc along with the right C4 and C5 lamina (Figure 2). The mass was 10 mm × 10 mm in size, and there was an appearance of cartilaginous tissue rather than a hematoma (Figures 2(a) and 2(b)). Decompression of the dura mater was confirmed intraoperatively after the removal of the mass. Hematoxylin-eosin staining showed cartilaginous tissue, there were no atypical cells, and the final diagnosis was chondromatous lesion (Figure 2(c)). The mass presented 2 years before, and we made a diagnosis of intraligamentous chondroma of the cervical spine. Postoperatively, the quadriparesis and MMT results improved, and he was able to resume walking. At the one-year follow-up, the patient's MMT results were almost normal and he could walk independently with a cane. No recurrence was observed on follow-up MRI (Figure 3).

## 3. Discussion

Soft tissue (extraskeletal) chondroma is a rare benign tumor. We searched “extraskeletal chondroma” in PubMed and there are 42 English papers. The location was various, hand and forearm [10–24], foot [25–31], head and neck [6, 32–37], knee [38–42], gynecologic area [43, 44], axilla [45], abdominal wall [46], and gluteal region [47]. The most common location is the hand and forearm. This tumor is thought to arise from the fibrous stroma of soft tissues rather than originating from mature cartilaginous or osseous tissue. Extraskeletal chondroma typically affects adults, usually between the ages of 30 and 60 years [27, 45].

We have described a very rare case of chondroma in the ligamentum flavum at the level of the cervical spine. A literature search in 2018 by Inoue et al. [8] identified 16 cases of chondroma in the cervical spine region, implying that our case is the 17th to be reported. Moreover, in our patient, the tumor was in the ligamentum flavum at the C4-C5 level. As mentioned earlier, there has only been 1 report of a chondroma arising in ligamentous tissue and involving the knee joint [9]. Therefore, a chondroma in the cervical ligamentum flavum would be rare indeed, and this case could be the first report.

In our patient, the symptoms had an acute onset and the tumor was located in the ligamentum flavum. We initially suspected that the mass was an intraligamentous hematoma. Tamura et al. [48] had previously reported a case of acute-onset quadriparesis caused by intraligamentous hematoma in the cervical spine. The clinical presentation in our patient was similar to that in their case, and there was no reason to suspect a tumor as the cause of his acute-onset quadriparesis. The tumor 2 years earlier was small and did not compress the spinal cord. The tumor had gradually increased in size in the intervening 2 years. Surgeons should be aware that patients with slow-growing benign tumors like chondroma could present with acute-onset quadriparesis.

It is unclear how chondroma can arise in a ligament or other soft tissue. It is well known that mesenchymal stem cells can differentiate to chondrocytes in vitro [49, 50]. Therefore, chondroma may arise from mesenchymal stem cells via some form of tumorigenesis [51]. Ligaments also contain mesenchymal stem cells, but the number is small and their clinical significance remains unclear [6]. This may explain why chondromas arising in ligamentous tissue are so rare.

## Figures and Tables

**Table 1:** Results of manual muscle testing.

Muscles tested	Right	Left
Deltoid	4	4
Biceps	3	5
Brachioradialis	3	5
Triceps	3-	5
Carpal flexors	2	5
Iliopsoas	3	4
Quadriceps	4	4
Tibialis anterior	2-	4
